# Validation of a Remote and Fully Automated Story Recall Task to Assess for Early Cognitive Impairment in Older Adults: Longitudinal Case-Control Observational Study

**DOI:** 10.2196/37090

**Published:** 2022-09-30

**Authors:** Caroline Skirrow, Marton Meszaros, Udeepa Meepegama, Raphael Lenain, Kathryn V Papp, Jack Weston, Emil Fristed

**Affiliations:** 1 Novoic Ltd London United Kingdom; 2 Center for Alzheimer Research and Treatment, Department of Neurology Brigham and Women’s Hospital Harvard Medical School Boston, MA United States; 3 Department of Neurology Massachusetts General Hospital Harvard Medical School Boston, MA United States

**Keywords:** neurology, memory, episodic, speech, psychometrics, reliability, validity, aging, elder, older adult, Alzheimer disease, mild cognitive impairment, mobile apps, mobile health, mHealth, smartphone, cognition, cognitive decline, cognitive impairment, development, validation, recall, memory, story, stories, observational study, acceptability, usability, semantic, cognitive test, speech, linguistic, mobile phone

## Abstract

**Background:**

Story recall is a simple and sensitive cognitive test that is commonly used to measure changes in episodic memory function in early Alzheimer disease (AD). Recent advances in digital technology and natural language processing methods make this test a candidate for automated administration and scoring. Multiple parallel test stimuli are required for higher-frequency disease monitoring.

**Objective:**

This study aims to develop and validate a remote and fully automated story recall task, suitable for longitudinal assessment, in a population of older adults with and without mild cognitive impairment (MCI) or mild AD.

**Methods:**

The “Amyloid Prediction in Early Stage Alzheimer’s disease” (AMYPRED) studies recruited participants in the United Kingdom (AMYPRED-UK: NCT04828122) and the United States (AMYPRED-US: NCT04928976). Participants were asked to complete optional daily self-administered assessments remotely on their smart devices over 7 to 8 days. Assessments included immediate and delayed recall of 3 stories from the Automatic Story Recall Task (ASRT), a test with multiple parallel stimuli (18 short stories and 18 long stories) balanced for key linguistic and discourse metrics. Verbal responses were recorded and securely transferred from participants’ personal devices and automatically transcribed and scored using text similarity metrics between the source text and retelling to derive a generalized match score. Group differences in adherence and task performance were examined using logistic and linear mixed models, respectively. Correlational analysis examined parallel-forms reliability of ASRTs and convergent validity with cognitive tests (Logical Memory Test and Preclinical Alzheimer’s Cognitive Composite with semantic processing). Acceptability and usability data were obtained using a remotely administered questionnaire.

**Results:**

Of the 200 participants recruited in the AMYPRED studies, 151 (75.5%)—78 cognitively unimpaired (CU) and 73 MCI or mild AD—engaged in optional remote assessments. Adherence to daily assessment was moderate and did not decline over time but was higher in CU participants (ASRTs were completed each day by 73/106, 68.9% participants with MCI or mild AD and 78/94, 83% CU participants). Participants reported favorable task usability: infrequent technical problems, easy use of the app, and a broad interest in the tasks. Task performance improved modestly across the week and was better for immediate recall. The generalized match scores were lower in participants with MCI or mild AD (Cohen *d*=1.54). Parallel-forms reliability of ASRT stories was moderate to strong for immediate recall (mean rho 0.73, range 0.56-0.88) and delayed recall (mean rho=0.73, range=0.54-0.86). The ASRTs showed moderate convergent validity with established cognitive tests.

**Conclusions:**

The unsupervised, self-administered ASRT task is sensitive to cognitive impairments in MCI and mild AD. The task showed good usability, high parallel-forms reliability, and high convergent validity with established cognitive tests. Remote, low-cost, low-burden, and automatically scored speech assessments could support diagnostic screening, health care, and treatment monitoring.

## Introduction

With the first disease-modifying treatment for Alzheimer disease (AD) now available [1], there is an increased need for broader screening and improved monitoring of disease progression and treatment response. Cognitive assessments are currently some of the least invasive and most cost-effective measures available for screening for AD and related impairments. Furthermore, they are supported for use as endpoints of treatment efficacy early in AD by key regulatory bodies, including the US Food and Drug Administration [2] and the European Medicines Agency [3].

However, many cognitive assessments are lengthy, require trained personnel to administer and score, and offer few parallel test variants, making them susceptible to practice effects. More importantly, test performance is measurably influenced by a range of state factors such as sleep [4], exercise [5], mood [6], and stress [7]. This variation can lead to inaccurate impression of improvement or decline over time [8]. Higher-frequency sampling can generate more stable and reliable estimates of constructs of interest by controlling for state effects [9] and delineating short-term cognitive fluctuations from longer-term changes associated with treatment response and disease progression [8].

Story recall is a cognitive testing paradigm used to assess verbal episodic memory and is commonly used to track AD-related decline, often as a component of cognitive composite tests [10-14]. Story recall is impaired in Alzheimer dementia [15], shows variable differentiation of individuals with mild cognitive impairment (MCI) from those that are cognitively unimpaired (CU) [16], and predicts progression from MCI to Alzheimer dementia [17].

Most story recall tests are administered in person and scored manually, but research has shown that scoring can be fully automated using natural language processing technologies [18]. This suggests that story recall tests could be administered in clinic at a lower cost and with reduced clinician time burden. Moreover, these tests may be suitable for use in remote assessment, provided that they are properly developed and validated and that test administration can be automated.

Although remote digital assessments are not new, the COVID-19 pandemic accelerated the need to adopt remote or hybrid clinical assessment or research methods [19,20]. Alongside advances in technology and connectivity, this has led to a growing interest in the use of personal digital devices to collect clinically informative data. Beyond this, digital health technologies can enhance inclusivity, improving access for people who experience mobility problems or those with financial, geographical, or time restrictions [21]. The continued drive toward remote assessment may be particularly important in older adults who are at a substantially increased risk in the pandemic [22]. Although holding promise for improving convenience and access, there are concerns about whether digital assessment methods are particularly challenging in this population, particularly for those with dementia or milder forms of cognitive impairment [23].

This study describes the Automatic Story Recall Task (ASRT), a remote, self-administered, and automatically scored test developed for repeated cognitive assessment, opening up opportunities for more nuanced longitudinal data analysis. Test characteristics were examined in participants who were CU, and individuals with MCI or mild AD. Participants were assessed repeatedly over 1 week. This study examined (1) the acceptability of remote ASRT assessment, (2) adherence to daily ASRT assessments, (3) parallel-forms reliability, (4) convergent validity with cognitive and clinical assessments, (5) task performance characteristics, and (6) the effect of daily internal state factors.

## Methods

### Recruitment

Participants were recruited from November 2020 to August 2021 in the United Kingdom (London, Guildford, Plymouth, and Birmingham) and the United States (Santa Ana, California). Research participants were enrolled if they were CU or diagnosed with MCI in the previous 5 years. In the UK study, participants diagnosed with mild AD in the last 5 years were also included. MCI due to AD and mild AD diagnoses were made according to the National Institute on Aging–Alzheimer’s Association core clinical criteria [24]. Participants were approached if they had undergone a prior amyloid beta positron emission tomography scan or a cerebrospinal fluid test (confirmed amyloid beta negative within 30 months or amyloid beta positive within 60 months). Eligibility was established by screening via video calls using a secure Zoom (Zoom Video Inc) link (UK study) or in-clinic assessment (US study), during which the Mini-Mental State Examination (MMSE) [25] was administered. For remote administration, no controls for potential environmental prompts to orientation questions (calendars, clocks, watches, etc) were implemented.

Inclusion criteria were as follows: age 50 to 85 years; MMSE raw score of 23 to 30 for participants with MCI or mild AD and 26 to 30 for CU; CU or clinical diagnosis of MCI or mild AD made in the previous 5 years; English as a first language; availability of a study partner for Clinical Dementia Rating scale (CDR) [26] semistructured interview; and access and ability to use a smartphone running an operating system of Android 7 or above, or iOS 11 or above.

Exclusion criteria were as follows: current diagnosis of general anxiety disorder, recent (6 month) history of unstable psychiatric illness, history of stroke within the past 2 years, or a documented history of transient ischemic attack or unexplained loss of consciousness in the last 12 months. Participants treated with medications for symptoms related to AD were required to be stabilized on these medications for at least 8 weeks before study entry and throughout the study. Participants with a current diagnosis of major depressive disorder (United Kingdom) or those with a current or 2-year history of major depressive disorder (United States) were excluded.

### Ethics Approval

This research was approved by the institutional review boards of the relevant research authorities (UK research ethics committee reference: 20/WM/0116; US Institutional Review Board reference: 8460-JGDuffy). Informed consent was obtained at the study site (United States) or electronically in accordance with the health research authority guidelines (United Kingdom). The studies are registered at ClinicalTrials.gov (NCT04828122 and NCT04928976).

### Procedure

#### Clinical Assessments

Participants completed clinical assessments via a secure Zoom link (United Kingdom) or in clinic (United States), completed with a trained psychometrician. The tests reported in this study are described in detail below.

The Wechsler Logical Memory (LM) Test “Anna Thompson” story variant evaluated the free recall of a story according to 25 predefined information units (a metric quantifying the amount of information recalled [27]) immediately after presentation and after a 30-minute delay. Variants presented included the original Wechsler Memory Scale (WMS) text for the US sample [28] and the WMS 3rd edition text for the UK sample [29]. Paraphrased answers were accepted for both text variants, and scoring was completed manually according to the instructions and in alignment with the administration and scoring guidelines. The immediate and delayed recall scores were obtained.

Cognitive tests incorporated in the Preclinical Alzheimer’s Cognitive Composite with semantic processing (PACC5) were administered. Tests were manually scored, and a mean *Z*-score was calculated as described previously [11]. The composite includes summary scores from five measures: (1) the MMSE [25], a global cognitive screening test; (2) LM Delayed Recall [28,29], a delayed story recall test; (3) Digit-Symbol Coding [30], a symbol substitution test; (4) the sum of free and total recall from the Free and Cued Selective Reminding Test [31], a multimodal associative memory test; and (5) category fluency (animals, vegetables, and fruits), a semantic memory test.

The CDR [26], a semistructured interview assessing the severity of cognitive symptoms of dementia, was completed with the participants and their study partners and scored based on the CDR–Sum of Boxes (CDR-SB) scales. The examiner was not blinded to the other assessments administered. In the US study, where participants had completed subtests of the PACC5 or CDR assessments within 1 month before the study visit, tests were not readministered, but the recent historical test results were used.

Participants completed the ASRT, a task constructed to elicit naturalistic speech within a closed domain. Prerecorded ASRTs were presented at a steady reading rate (approximately 140 words per minute) by a British male speaker. Parallel stimuli included 36 stories: 18 short stories (119 words per story, SD 4.83) and 18 long stories (224 words per story, SD 14.86). Task characteristics are presented in Table S1 in Multimedia Appendix 1, showing that stories incorporate a range of themes and are balanced for key linguistic and discourse metrics. During clinical assessments, 3 long ASRT stories were administered consecutively. After each story was presented, participants were asked to immediately retell the story in as much detail as they could remember. Recall of the same stories, in the same order, was tested again after a delay.

During clinical assessments, participants were supported with installing the Novoic mobile app (“the app”) on their own smartphone device and were shown how to use it. Participants were reimbursed for their participation at the end of the study visit and before remote assessments (£65 [US $86] for United Kingdom participants and US $75 for US participants). No threshold for use during remote follow-up was required for participants to be fully remunerated.

#### Remote Assessments

Participants were encouraged to complete optional unsupervised self-assessments (<30 minutes in length) on the app daily for up to 8 days following the study visit. Assessments included ASRTs and other remote speech tasks not reported here (verbal and category fluency assessments, reading tasks, picture description, and procedural discourse tasks) as well as remote questionnaires. ASRTs were administered at the beginning of each assessment, with the order, inclusion, and administration of other tests varying by day.

Distinct assessment components (ASRTs [+fluency tasks as appropriate], questionnaires, and other tasks) were divided so that participants, once completing one component, were informed of their progress and given the opportunity to continue. This meant that participants could take breaks between assessment components. All ASRTs were administered within one of these assessment components, without interruption. If and where participation was interrupted because of other factors (distraction, etc), individual audio tasks administered were not repeated, but participants were able to continue with the following part of the assessments.

Remote ASRTs were administered daily, in threes (triplets) and at the beginning of each assessment session. The ASRT stories administered on the first day of remote assessment were identical to those administered in the clinical supervised assessment on the prior day, to allow for the evaluation of practice effects (not reported here). The remainder of the ASRT stories, presented from day 2 of remote assessment onward, were novel and administered only once.

After each story was presented, participants were asked to immediately retell the story in as much detail as they could remember. Recall of the same stories, in the same order, was tested again after a delay. The schedule included delayed recall after completion of all immediate recalls or after completion of brief distractor tasks (fluency tasks: category or verbal fluency), with test administration varying by day (shown in Table S2 in Multimedia Appendix 1). Recordings of spoken responses were automatically started by the app following instructions and manually stopped by the participants. These were recorded as audio files on participants’ personal smart devices and automatically uploaded to a secure server.

Due to participant-initiated feedback of high burden (that the remote assessments were too long and tiring), the assessment schedule was changed partway through the study. The new schedule implemented shorter stories and reduced the number of additional assessments following ASRTs (not reported here). However, ASRTs continued to be administered daily at the start of each assessment. Simultaneously, the number of days of remote assessment was increased from 7 days to 8 days to spread out assessments and reduce the daily burden. Details are provided in Table S2 in Multimedia Appendix 1.

Daily state effects were assessed at the end of each remote assessment via a 4-item self-report questionnaire asking how participants were feeling that day (current mood, quantity of sleep, mind-wandering, and effort), scored on a 7-point response scale from “much worse/less than usual” to “much better/more than usual.” App and task usability were assessed via a self-report questionnaire on day 2 (initial assessment schedule) or day 5 (revised assessment schedule). Usability questionnaires asked participants to report technical difficulties experienced during assessments, whether technical difficulties prevented them from completing the assessments, how easy it was to use the app, and how interesting the tasks were. Questionnaires are shown in Tables S3 and S4 in Multimedia Appendix 1.

### Statistical Analysis

ASRT task responses were transcribed using Google’s speech-to-text [32] automatic speech recognition system, using an enhanced speech recognition model (the “video” model, suitable for recordings that may contain background noise). All task responses were also transcribed manually by following a standardized procedure, which included transcription of commentary, filled pauses, and partial words. The word error rate (WER) of the automatic transcript was calculated using the HuggingFace package [33], as the average number of errors per manual transcript word. This was calculated after removing punctuation, setting all text characters to lower case, and removing filled pauses and partial words from the transcripts before comparison.

Transcription was followed by automated textual analysis completed using a generalized match (G-match) score. G-match was computed in Python as the weighted sum of the cosine similarity between the embeddings of original ASRT text and the transcribed retellings, providing an automatic quantitative evaluation of similarity across the 2 texts. G-match provides an index of the proportional recall for each story, with potential scores ranging from 0 to 1 (hypothetically perfect performance). Mean G-match per triplet was also computed. The underlying representations of the model are based on a pretrained BERT model [34], which is a large language model pretrained on a corpus of more than 3000 million words, to produce generalized representations of language and how it is used.

Further analysis was performed using the statistical software package R v.4.0. Data were assessed for normality, followed by parametric and nonparametric analyses as appropriate. Adherence was defined as engaging with at least one ASRT story per day. Adherence patterns were examined with logistic regression models, predicting adherence at immediate and delayed recall, in relation to participant group, demographics, assessment day, and schedule. A large proportion of participants completed only 7 days of remote assessments, and longitudinal analysis of adherence was therefore limited to assessments on days 1 to 7. Participants were included as random effects. Demographics (sex, age, and years of education), assessment days (1-7), research schedule (schedule 1 or schedule 2), and participant group (CU and MCI or mild AD) were included as fixed factors.

The parallel-forms reliability of ASRTs was examined using pairwise correlational analysis. Only ASRT stories administered across both test schedules were analyzed, maintaining comparable sample sizes across comparisons and allowing for testing within the MCI or mild AD and CU subgroups. The convergent validity of these same ASRT stories was examined in relation to LM, PACC5, and CDR-SB. Analyses were repeated using the mean G-match score per triplet. Spearman rank correlation coefficients are reported throughout to maintain the consistency and comparability of reporting.

Task performance differences between groups, task administration variations, and change over time were modeled using longitudinal linear mixed-effects models. Data analyzed were restricted to remote assessment days 2 to 7, when assessments were novel and administered to all participants. The mixed model analysis included G-match as the response variable, and fixed effects of participant group, remote assessment days (2-7), order (1st, 2nd, or 3rd ASRT presented), long or short stories, and immediate or delayed recall. Demographics (age, sex, and education) were included as additional fixed effects. A random effect of participant with random slope and intercept was specified. Cohen *d* effect sizes for multilevel model objects were calculated using the lme.dscore command in the package EMAtools.

Analyses were repeated with the mean G-match per triplet, with equivalent random and fixed effects specifications, except for the story order, which was not included. The covariation of mean ASRT task performance across triplets with self-reported daily state was then examined by additionally incorporating fixed effects of self-reported mood, sleep, effort, and mind-wandering into the above model. The assumptions of all regression models were investigated by examining the distribution and patterns of residuals versus fitted values.

Group differences and effect sizes were also evaluated for traditional cognitive tests completed with a trained psychometrician via Zoom or in person during clinical assessments. Comparisons were only carried out for tests that were not directly or indirectly part of the study selection criteria (Digit-Symbol Coding, the Free and Cued Selective Reminding Test, and category fluency), thereby excluding MMSE (direct selection criterion), the LM Delayed Recall, and PACC5 (indirect). As participants were recruited from prior completed trials, in some of which performance thresholds on the LM delayed recall contributed to the MCI and mild AD group inclusion criteria, LM and PACC5 (of which the LM is a component) were not evaluated. The test distributions of traditional cognitive assessments were evaluated for normality, followed by parametric or nonparametric tests, as appropriate.

## Results

### Participants

A total of 200 participants, 67 from the US study and 133 from the UK study, were recruited and completed the clinical assessment protocol. In total, 75.5% (151/200) of the participants completed at least one remote ASRT. Older participants (*r*=−0.15; *P*=.03), those with lower MMSE scores (*r*=−0.26; *P*<.001), and those with MCI or mild AD (33/106, 31.1% MCI or mild AD, compared with 16/94, 17% CU; χ^2^_1_=5.4; *P*=.02) more often did not complete any remote assessments. There were no differences in sex ratio (χ^2^_1_=0.4; *P*=.50) or years of education (*r*=−0.01; *P*=.87) between participants who contributed at least one remote assessment and those who did not.

Demographic information of the participants providing remote data are presented in Table 1. In this sample, the MCI or mild AD and CU groups did not differ with respect to age, years of education, sex, or amyloid status. The US study included proportionally more participants with cognitive impairment (22/34, 65% with MCI) than the UK sample (51/117, 43.6% with a diagnosis of MCI or mild AD). The MCI or mild AD group included a minority of participants with a diagnosis of mild AD (10/73, 14%), all recruited into the UK sample as per the inclusion criteria. A detailed breakdown of the sample characteristics by US and UK studies is provided in Table S5 in Multimedia Appendix 1.

**Table 1:** Participant demographic characteristics of cognitively unimpaired participants and participants with MCI^a^ or mild AD^b^.

			Group					Statistical values			
			Cognitively unimpaired (n=78)		MCI or mild AD (n=73)		Test statistic			*P* value	
**Sex, n (%)**								χ^2^_1_=0.3			.61
	Female	47 (60)		41 (56)							
	Male	31 (40)		32 (44)							
**Country of residence, n (%)**								χ^2^_1_=4.7			.03
	United Kingdom	66 (85)		51 (70)							
	United States	12 (15)		22 (30)							
**Testing schedule, n (%)**								χ^2^_1_=7.0			.008
	Schedule 1	40 (51)		22 (30)							
	Schedule 2	38 (49)		51 (70)							
**Amyloid beta status, n (%)**								χ^2^_1_=0.8			.36
	Amyloid negative	38 (49)		41 (56)							
	Amyloid positive	40 (51)		32 (44)							
Years of education, mean (SD)			15.24 (3.37)		15.06 (2.80)		*r*=−0.05			.57	
Age (years), mean (SD)			70.37 (4.35)		69.58 (7.30)		*r*=−0.01			.91	
MMSE^c^, mean (SD)			28.92 (1.15)		27.00 (2.07)		*r*=0.50			<.001	

^a^MCI: mild cognitive impairment.

^b^AD: Alzheimer disease.

^c^MMSE: Mini-Mental State Examination.

### Usability

**Figure 1:**
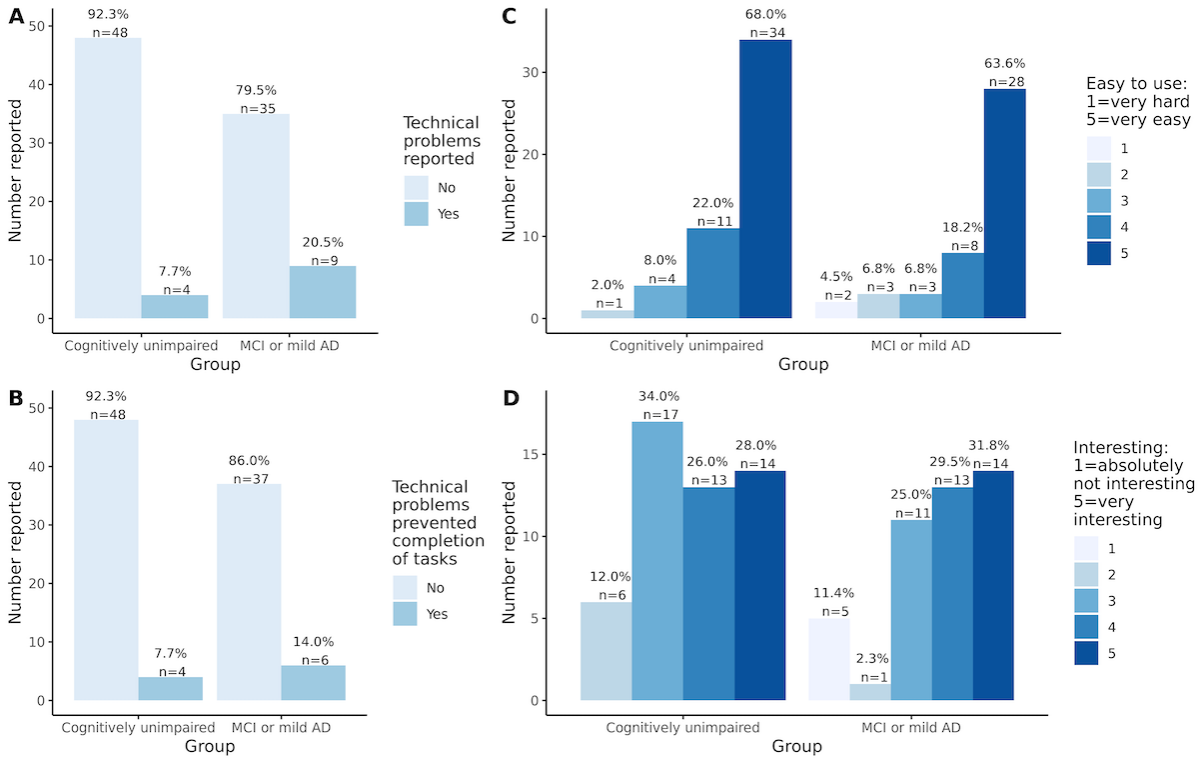
Responses to usability questionnaire: (A) technical problems reported, (B) rate at which technical problems prevented completion of tasks, (C) ease of use of app, and (D) interest in tasks completed. AD: Alzheimer disease; MCI: mild cognitive impairment.

Usability questionnaires were completed by 63.6% (96/151; CU: n=52 and MCI or mild AD: n=44) of the participants who completed remote assessments (Figure 1). Those completing usability questionnaires did not differ with respect to education level (*r*=−0.02; *P*=.78), age (*r*=−0.12; *P*=.14), or MMSE scores (*r*=−0.08; *P*=.32) compared with those who engaged in remote assessments but did not complete usability questionnaires. There was also no difference in the male to female ratio (χ^2^_1_=0.1; *P*=.75) or the ratio of CU participants to participants with MCI or mild AD (χ^2^_1_=0.7; *P*=.41) who did and did not complete usability questionnaires.

In total, 8% (4/52) of CU participants and 20% (9/44) of participants with MCI or mild AD reported technical difficulties. Where technical difficulties were encountered, most participants reported that these did not prevent them from completing the assessments, with no group differences (χ^2^_1_=3.3; *P*=.07 and χ^2^_1_=1.0; *P*=.32, respectively for technical difficulties reported, and inability to complete assessments). Most participants responded that the app was easy to use and that the task was reasonably interesting, with no group differences (*r*=−0.08; *P*=.47 and *r*=−0.04; *P*=.70, respectively for ease of use and interest in tasks).

### Adherence

**Figure 2:**
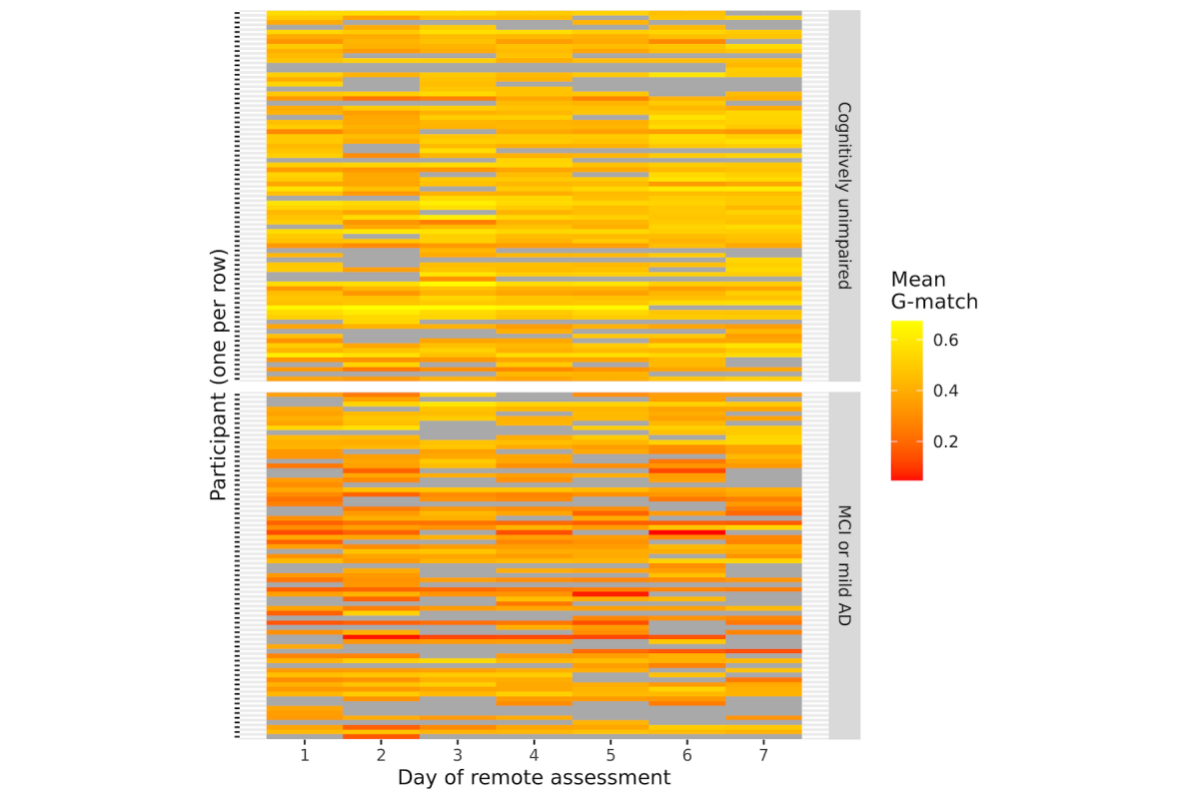
Adherence and task performance heat map for generalized match (G-match) in immediate recall trials. G-match is an automated measure of recall performance (refer to the Methods section). Results are plotted across individual days of remote assessment for 151 participants who completed at least one assessment. Each participant is represented by a row, missing data are shown in gray, and mean G-match across the Automatic Story Recall Task triplets is shown in color (red=low recall and yellow=high recall). AD: Alzheimer disease; MCI: mild cognitive impairment.

Participants with MCI or mild AD completed fewer remote assessments than CU participants (adherence for immediate recall: 64.5% vs 77.5%; delayed recall: 61.5% vs 77.3%; Figure 2). Group differences were confirmed by mixed logistic regression analyses (immediate recall estimate=−0.97; *P*=.01 and delayed recall estimate=−0.84; *P*=.02). Adherence did not change over the assessment days (immediate recall estimate=−0.04; *P*=.34 and delayed recall estimate=−0.07; *P*=.11), but lower adherence to delayed recall was observed for the revised test schedule (estimate=−0.86; *P*=.03). Adherence was not associated with sex and education (all *P*>.20), but younger participants completed more immediate recall assessments (immediate recall estimate=−0.07; *P*=.02 and delayed recall estimate=−0.06; *P*=.06).

Figure 2 shows a heat map of the adherence patterns and task performance. In this figure, each participant is represented by a row, and task response and performance over the days of assessment are shown in colored blocks along the x-axis. Task performance is shown in color, with red to yellow grading representing low to high G-match scores. Missing data are shown in gray. This figure reflects the results reported above, with higher adherence in the CU group and no clear decline in adherence over the assessment period.

### Transcription Accuracy

The average WER for participant recordings of automatic transcripts compared with manual transcripts was 0.11. The average WER differed across participant groups, with WER=0.09 in CU participants and WER=0.13 in participants with MCI or mild AD (t_108.1_=−3.81; *P*<.001; Cohen *d*=0.63).

### Task Characteristics

**Figure 3:**
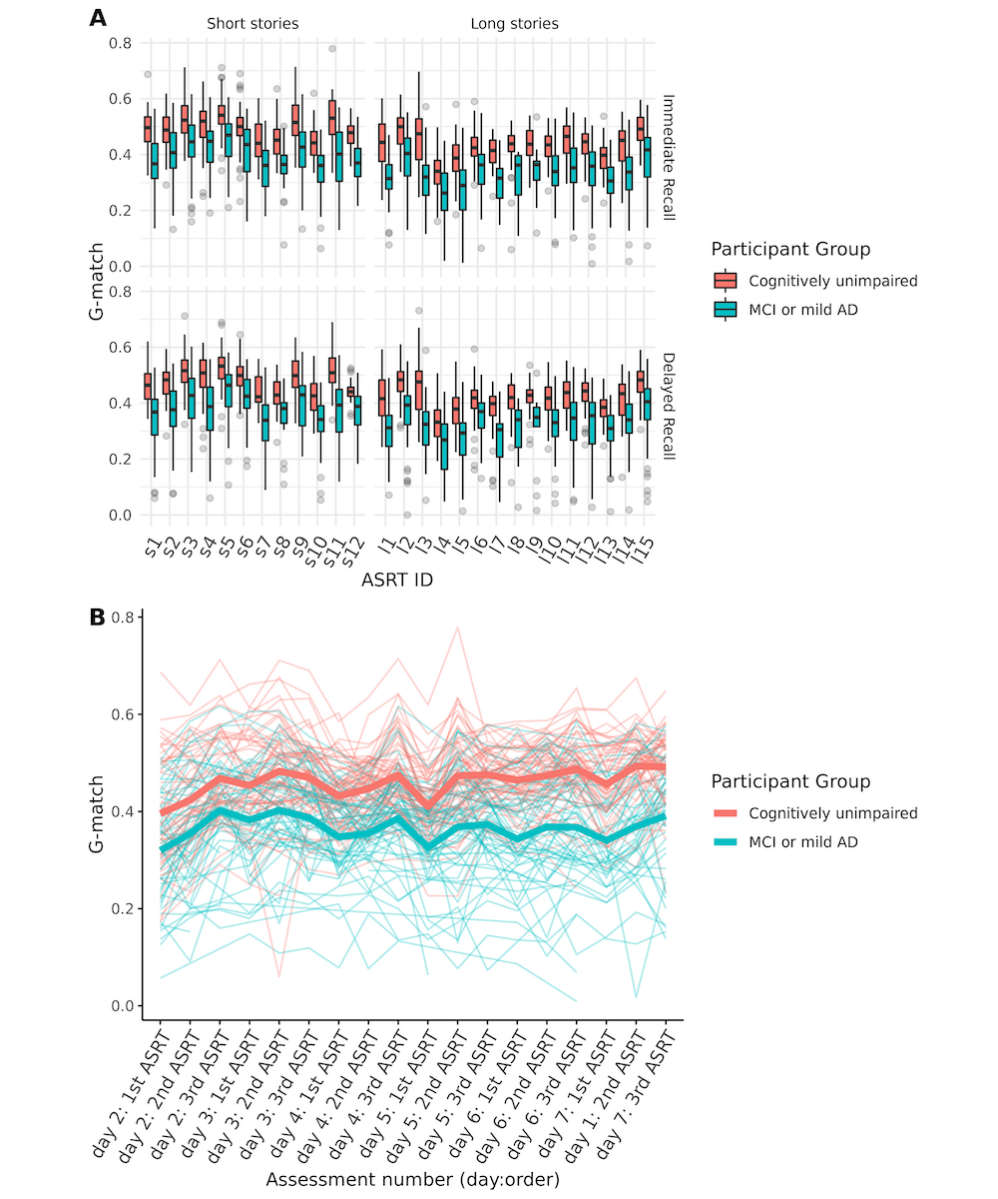
Generalized match (G-match) over repeated assessments: (A) boxplots of G-match for individual Automatic Story Recall Task stories split by short and long stories horizontally and by immediate and delayed recalls vertically and (B) average G-match (immediate recall) over individual assessment days (2-7 and immediate recall) and testing order. Group means are displayed with the thick lines, and individual participant trajectories across assessments and days are shown with paler, thinner lines. AD: Alzheimer disease; ASRT: Automatic Story Recall Task; MCI: mild cognitive impairment.

G-match for ASRTs and triplets showed good psychometric properties. Data generated showed no ceiling or floor effects (Figure 3A; Figures S1-S4 in Multimedia Appendix 1). Task performance characteristics are provided in Tables S6-S8 in Multimedia Appendix 1.

### Parallel-Forms Reliability

**Figure 4:**
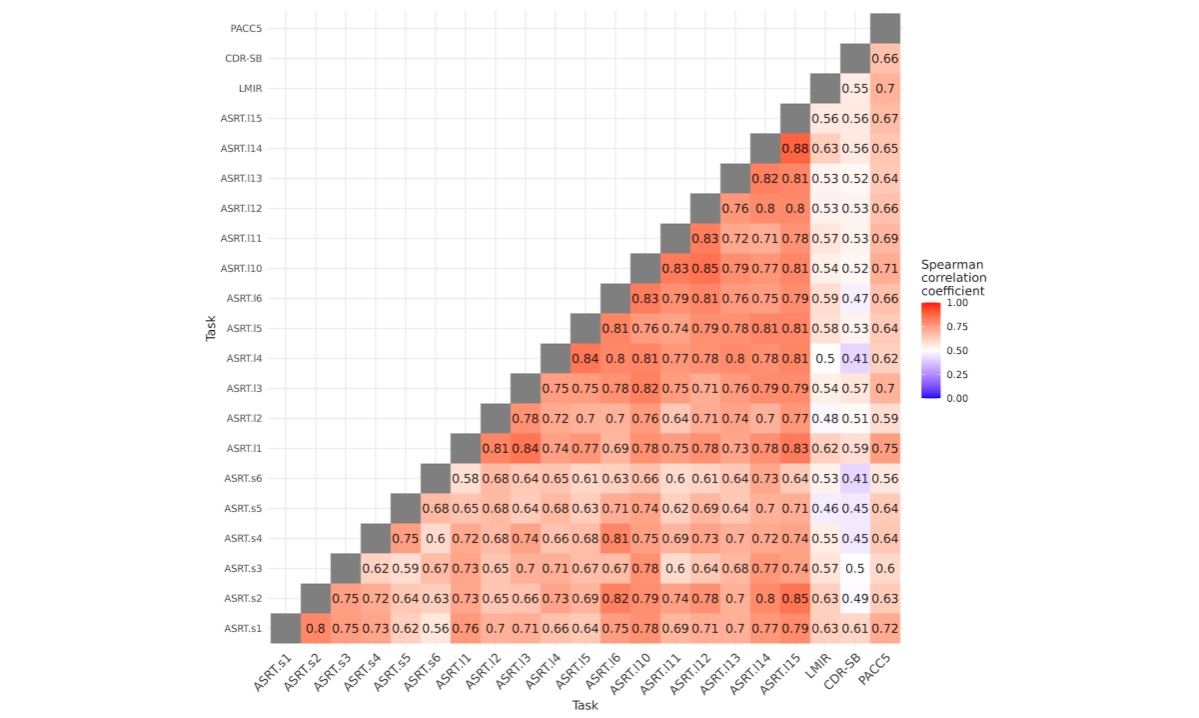
Parallel forms reliability and convergent validity of Automatic Story Recall Task (ASRT) stories at immediate recall. ASRTs are denoted with s (short) and l (long), followed by the story number (refer to Table S1 in Multimedia Appendix 1). Correlations with other assessments are displayed (Wechsler Logical Memory Test–Immediate Recall [LMIR], Clinical Dementia Rating scale–Sum of Boxes [CDR-SB], and Preclinical Alzheimer’s Cognitive Composite with semantic processing [PACC5]). The sign for the CDR-SB correlation is reversed for consistency. Correlation coefficients derived from between 75 and 116 participants, depending on adherence patterns.

Parallel-forms reliability for individual ASRT stories at immediate recall are presented in Figure 4. Equivalent figures for delayed recall, separated by clinical group, are presented in Figures S5-S9 in Multimedia Appendix 1. Correlation matrices for triplets separated by immediate and delayed recall, and clinical groups, are shown in Figures S10-S12 in Multimedia Appendix 1.

Correlation coefficients in the full sample were moderate to strong for immediate recall (rho range=0.56-0.88; mean 0.73) and remained so after restricting analyses to participants with MCI or mild AD (rho range=0.31-0.87; mean 0.65) and CU participants (rho range 0.39-0.85; mean 0.65). Similarly, correlations between parallel ASRT stories were moderate to high for delayed recall (full sample: rho range=0.54-0.86; mean 0.73) and remained so when restricting analyses to participants with MCI or mild AD (rho range=0.37-0.88; mean 0.65) and CU participants (rho range=0.32-0.83; mean 0.64).

Parallel-forms reliability was higher when examined for mean scores obtained across triplets (immediate: rho range=0.77-0.88, mean 0.83; and delayed: rho range=0.76-0.89, mean 0.85), remaining consistently high in MCI or mild AD (immediate: rho range=0.57-0.88, mean 0.73; and delayed: rho range=0.60-0.89, mean 0.75) and CU subgroups (immediate: rho range=0.67-0.83, mean 0.76; and delayed: rho range=0.68-0.85, mean 0.77).

### Convergent Validity

ASRT task performance correlated moderately with other cognitive and clinical measures (LM, CDR-SB, and PACC5) in the full sample across both immediate and delayed recalls (Figure 4). The mean correlation coefficients between immediate recall ASRTs with LM-immediate recall, PACC5, and CDR-SB were rho=0.56, 0.65, and 0.51, respectively. The mean correlation coefficients between ASRTs with LM-delayed recall, PACC5, and CDR-SB were rho=0.54, 0.66, and 0.50, respectively. Analysis results and figures for delayed recall and results separated by participant group are provided in Figures S5-S9 in Multimedia Appendix 1. Correlation coefficients remained in the moderate range after restricting analyses to participants with MCI or mild AD but were typically lower in CU participants. Correlations between ASRT triplets and other cognitive tests are provided in Figures S10-S12 in Multimedia Appendix 1.

### Task Performance Comparison Between Groups

The longitudinal mixed models are presented in Table 2, with similar results for individual ASRTs and triplets. Task performance improved across the week, with a modest linear daily improvement in G-match by assessment day. There was an effect of group with lower scores in the MCI or mild AD group for both individual stories and triplets, with an effect size of Cohen *d*=1.54. G-match was higher for immediate recall and shorter stories and higher for the latter ASRTs administered within each triplet. Demographics were not associated with task performance. Longitudinal data are displayed in Figure 3B, showing within- and between-subject variability.

**Table 2:** Effects of task characteristics, participant group, and demographics on task performance metrics as estimated by longitudinal mixed models. For binary predictors (sex, ASRT^a^ length, and recall type) the reference category is listed first.

	G-match individual stories		G-match triplets	
	Estimate (SE)	*P* value	Estimate (SE)	*P* value
Intercept	0.53 (0.08)	<.001	0.57 (0.08)	<.001
Group (Group 1: CU^b^, Group 2: MCI^c^ or mild AD^d^)	−0.11 (0.01)	<.001	−0.11 (0.01)	<.001
Assessment day	0.005 (0.001)	<.001	0.005 (0.001)	<.001
Recall type (immediate and delayed)	−0.02 (0.001)	<.001	−0.02 (0.002)	<.001
ASRT length (short and long)	−0.04 (0.003)	<.001	−0.04 (0.003)	<.001
ASRT order of presentation (1,2, and 3)	0.02 (0.001)	<.001	—^e^	—
Sex (female and male)	−0.02 (0.01)	.08	−0.02 (0.01)	.07
Education (years)	0.0004 (0.002)	.83	0.0003 (0.002)	.85
Age (years)	−0.002 (0.001)	.12	−0.002 (0.001)	.13

^a^ASRT: Automatic Story Recall Task.

^b^CU: cognitively unimpaired.

^c^MCI: mild cognitive impairment.

^d^AD: Alzheimer’s disease.

^e^Fixed effect not included in model.

After incorporating self-report assessments into the mixed model predicting G-match for triplets, the models revealed a significant effect of mood (estimate=0.007; SE 0.002; *P*<.001) and mind-wandering (estimate=−0.007; SE 0.002; *P*<.001), with better daily mood and lower mind-wandering associated with better daily task performance.

### Comparison With Traditional Neuropsychological Tests

Traditional neuropsychological tests administered in person during in-clinic assessments were also predictive of MCI or mild AD diagnostic status, with large effect sizes identified: Digit-Symbol Coding: t_82_=5.40, *P<*.001, Cohen *d*=1.07; category fluency total score: *t t*_148_=7.16, *P*<.001, Cohen *d*=1.17; and the sum of free and total recall from the Free and Cued Selective Reminding Test: t_108_=5.56, *P*<.001, Cohen *d*=1.01.

## Discussion

### Principal Findings

This study indicates that daily unsupervised and self-administered speech-based testing is acceptable and feasible for older participants with and without cognitive impairment. Participants engaged in daily optional assessments with moderate levels of adherence. There was no observable reduction in adherence levels over a weeklong period of assessment. The participants experienced infrequent technical problems and reported that the tests were easy to use and reasonably interesting.

Results indicate that remote automatic test administration and autoscoring of story recall can provide sensitive cognitive measurement in at-risk populations. The ASRT G-match, an automatically scored measure of proportional recall, showed consistent differences in task performance between cognitively healthy participants and those with MCI or mild AD. Separation in task performance between diagnostic groups was consistent across the assessment period and across individual ASRT stories (Figure 3), showing a strong effect size for differentiating CU participants from those with MCI or mild AD (Cohen *d*=1.54), while controlling for age, education, and sex. The equivalent area under the receiver operating characteristic curve was 0.86, based on previously published equivalence tables [35].

The effect size for ASRTs is larger than that seen for a range of traditional cognitive tests typically administered in person and under supervision. Comparisons with the LM delayed recall and PACC5 were not made, as participants in this study were recruited from prior trials in which test performance on LM delayed recall constituted part of the trial inclusion criteria for patients with MCI or mild AD, which would likely inflate effect sizes for these tests.

The ASRTs discrimination between clinical groups reported here outperforms those previously reported for differentiating CU individuals from those with MCI using other traditional cognitive tests administered in person and in the clinic, such as the MMSE (Cohen *d*=0.69), the 6-Item cognitive impairment test (Cohen *d*=0.65), and Addenbrooke’s Cognitive Examination-Revised (Cohen *d*=0.73), albeit with similar results reported previously for the Montreal Cognitive Assessment battery (Cohen *d*=1.45) [36]. The test also performs well in comparison with the Cogstate brief battery, when administered remotely and unsupervised, where effect sizes for differences between MCI and CU groups in subtests range from Cohen *d*=0.22 to Cohen *d*=0.62 [37].

Although the mixed clinical group examined in this analyses limits direct comparison with previously published metrics in subjects with MCI only, the mild AD group in this study comprised only a small proportion (10/73, 14%) of those contributing to the MCI or mild AD group. After excluding participants with mild AD from the linear mixed model analysis, this yielded an effect size of Cohen *d*=1.45 (equivalent area under the curve=0.85 [35]) for the difference between the CU participants and participants with MCI.

ASRT stimuli are carefully designed and balanced for key linguistic and discourse metrics, including the number of words, number of sentences, number of dependent clauses, mean sentence length, and ratio of dependent clauses to t-units (the number of shortest grammatically complete units into which a string of written or spoken language can be partitioned). This balancing of the stimuli is also reflected in good parallel-forms reliability between ASRT parallel stimuli, which is consistently high across immediate and delayed recall and with analyses constrained to clinical subgroups (MCI or mild AD and CU). The ASRT analysis pipeline also has significant advantages for test-retest reliability and parallel-forms reliability, as text similarity is evaluated in the same way every time, producing a standardized scoring system across the parallel test forms. A more objective quantification of text similarity, using a large language corpus for training, removes some of the more arbitrary features common to story recall task scoring, in which specific paraphrases are accepted, and the size of information units shows some variability [29].

Repeated exposure to the test stimuli may lead to unwanted practice effects that reduce the validity of the test as a measure of new learning, with research also showing differential practice effects across clinical diagnostic groups for tests such as list learning tests and LM [38]. Practice effects may be particularly important when considering where the same story recall stimuli are used repeatedly in longitudinal research or clinical monitoring or for diagnostic thresholding as cut-offs for research studies or clinical trials [16,39]. Other available story recall tests typically have a limited range of parallel forms.

The number of available parallel forms of the ASRT test allows for a higher-frequency (daily) assessment over a shorter period without test repetition, such as that carried out in this study. Alternatively, tests could be administered at larger intervals (weekly, monthly, or longer) to evaluate longer-term changes with little or no repetition of stimuli, thereby likely reducing practice effects.

Although alternate test variants can help reduce practice effects, they do not completely correct for retesting, which can be modified by repeated exposure to the task and greater familiarity with the test structure or method [40]. In this study, despite novel stimuli being presented during each assessment, test scores improved modestly during the week, indicating that increased familiarity with the app, testing procedure, and test structure resulted in a subtle improvement over time. Task performance improvements over the weeklong assessment period were modest (with an estimated daily change in G-match of only 0.4% of the initial intercept estimate value). The improvement in test performance, in combination with the absence of adherence changes over the course of the study, did not indicate any strong fatigue effects.

ASRT tests correlated moderately with a well-established test of verbal episodic memory, cognitive composites, and clinician-reported outcomes, indicating acceptable convergent validity, and with results comparable with, or better than, other studies of computerized or unsupervised remote assessments [41-43]. Correlations with LM and clinician-reported outcomes were in the moderate range, with lower correlation coefficients linked to test invariance owing to ceiling- or floor-level performance on these traditional clinical and neuropsychological assessments in CU individuals.

Task performance also varied with aspects of study design, with stories administered later in triplets delivering a more comprehensive recall. These effects appear to lead to greater variation between individual ASRT stories but are averaged out when the G-match is examined across story triplets. Analysis of story triplets showed higher parallel-forms reliability between the ASRTs administered and analyzed in threes, albeit with broadly unchanged differences in group performance. Task performance, as measured with G-match, was typically higher for shorter stories, indicating that responses more comprehensively covered the story source text where participants were asked to recall less material.

This study also showed within-subject variation in task performance, in part reflecting the measured effects of state factors on cognitive performance, in particular daily mood and effort. Variation from within-subject differences can make it challenging to differentiate clinical change from measurement error [8], and higher-frequency assessments carried out longitudinally can help generate more reliable estimates of cognitive function and change. Repeated measurements allow these state effects to be concurrently measured and included or controlled for in the longitudinal analyses.

### Limitations

To meet the eligibility criteria, participants were required to be able to use and access a smartphone. This may have biased the sample by overselecting those with higher familiarity with technology. Older and more cognitively impaired participants were less likely to contribute to the remote study component, and when they engaged in remote assessments, they contributed less frequently. However, the adherence statistics presented here reflect participants’ engagement in optional assessments, which may have differed had these been compulsory. Many home testing options require at least a modest level of technological fluency that some older adults may find challenging, challenges that may be compounded by cognitive impairment or comorbidities [44].

Therefore, the data presented may not reflect task performance in more impaired individuals or those with lower levels of technological familiarity. Assessments under supervision, either in the clinic or during a telemedicine visit, allowing for provision of additional support where required, could be better suited to more impaired individuals.

By collecting usability data during remote assessments, we were able to establish that most participants did not experience any technical problems and that the app was generally easy to use. However, more detailed qualitative feedback on the type of usability and any technical issues was not collected. Further evaluation of the nature of these difficulties is required. This information can be used to improve the user interface and participant engagement with remote assessments.

In response to participants’ and study centers’ feedback on the high participant burden of the initial test schedule, the testing schedule was altered in the middle of the study to reduce burden, thereby limiting the data available for certain ASRT test variants.

The design of the study makes it difficult to differentiate between the effects of individual stories themselves (ie, which ASRT story was used) and the effects of study design, such as the test order or day of assessment. Future studies may benefit from adopting a randomized design, with ASRTs randomly selected and allocated to different testing instances to derive test performance metrics independent of these additional confounders. For longitudinal studies, either short or long stories should be adopted to improve the consistency of test scores over time and help better characterize cognitive change.

We found differences in WER when comparing automated and manual transcripts of CU participants and those with MCI or mild AD, indicative of the differential intelligibility of speech or recording quality in these 2 groups. Differences in the performance of automatic transcription will impact the analysis further along in the analysis pipeline, indicating that group differences in scores likely reflect not only group differences in proportional recall but may also incorporate speech intelligibility and participant’s device use characteristics. However, these effects warrant further investigation.

The participants included in this study constituted a select sample. The sample was selected to exclude patients with concurrent neurological and mental health conditions. They were recruited from prior clinical trials completed in the United States and the United Kingdom and reflect a group of individuals who are actively engaged in clinical research. The participants lacked racial diversity (with most of the sample identifying as White and with only 2.6% [N=4] with Asian, Black, African, or African American background). Replication is now needed in more clinically and demographically heterogeneous samples.

### Overview and Future Directions

The recent Food and Drug Administration approval for the first disease-modifying treatment for people at risk of developing AD highlights the importance of adequate screening and early detection as well as the importance of monitoring treatment response. Briefer, convenient, and lower-burden daily assessments may provide more reliable data to evaluate disease progression or treatment response than lengthy one-off assessments [9]. Brief digital assessments completed at home and repeatable over time could improve access to AD screening compared with current clinical standards, which typically require clinical visits and extensive neuropsychological assessment.

This study showed that brief, remotely administered, and automatically scored ASRTs are sensitive to early cognitive impairments commonly identified through more extensive clinical assessments. The tests showed good properties for repeated administration and convergent validity with established tests of episodic memory, cognitive composites, and clinician-reported outcomes (CDR-SB). The test showed good acceptability and usability for older adults with and without cognitive impairment. Furthermore, owing to the automatic administration and scoring of ASRTs, this test presents a minimal administrative burden, requiring no trained personnel or specialist equipment.

Speech is instrumental in daily functioning and a natural response modality for participants to use in response to current smart devices, such as smartphones. Speech responses are also a common component of cognitive tests; however, data generated in these tests, including those reported in this study, often relate simple pass or fail characteristics of response accuracy. New metrics using audio- and text-based artificial intelligence models to target other changes measurable in speech data (acoustic [45,46], semantic [47-49], and linguistic [46]) in early-stage AD could further leverage the information content of ASRTs, developing a new class of powerful, fully automated speech biomarkers.

## Appendix

Multimedia Appendix 1 Supplementary information, tables, and figures for daily remote administration of the Automatic Story Recall Task.

## Abbreviations

AD: Alzheimer disease
ASRT: Automatic Story Recall Task
CDR: Clinical Dementia Rating scale
CDR-SB: Clinical Dementia Rating scale–Sum of Boxes
CU: cognitively unimpaired
G-match: generalized match
LM: Logical Memory
MCI: mild cognitive impairment
MMSE: Mini-Mental State Examination
PACC5: Preclinical Alzheimer’s Cognitive Composite with semantic processing
WER: word error rate

## Data Availability

Speech data are identifying and cannot be shared; however, all quantitative data produced in this study are available upon reasonable request to the authors.
